# Oral and Anal Vaccination Confers Full Protection against Enteric Redmouth Disease (ERM) in Rainbow Trout

**DOI:** 10.1371/journal.pone.0093845

**Published:** 2014-04-04

**Authors:** Kasper Rømer Villumsen, Lukas Neumann, Maki Ohtani, Helene Kragelund Strøm, Martin Kristian Raida

**Affiliations:** Research group of Fish Diseases and Immunology, Section of Veterinary Clinical Microbiology, Department of Veterinary Disease Biology, Faculty of Health and Medical Sciences, University of Copenhagen, Copenhagen, Denmark; University of Padova, Medical School, Italy

## Abstract

The effect of oral vaccines against bacterial fish diseases has been a topic for debate for decades. Recently both M-like cells and dendritic cells have been discovered in the intestine of rainbow trout. It is therefore likely that antigens reaching the intestine can be taken up and thereby induce immunity in orally vaccinated fish. The objective of this project was to investigate whether oral and anal vaccination of rainbow trout induces protection against an experimental waterborne infection with the pathogenic enterobacteria *Yersinia ruckeri* O1 biotype 1 the causative agent of enteric redmouth disease (ERM). Rainbow trout were orally vaccinated with AquaVac ERM Oral (MERCK Animal Health) or an experimental vaccine bacterin of *Y. ruckeri* O1. Both vaccines were tested with and without a booster vaccination four months post the primary vaccination. Furthermore, two groups of positive controls were included, one group receiving the experimental oral vaccine in a 50 times higher dose, and the other group receiving a single dose administered anally in order to bypass the stomach. Each group was bath challenged with 6.3×10^8^ CFU/ml *Y. ruckeri*, six months post the primary vaccination. The challenge induced significant mortality in all the infected groups except for the groups vaccinated anally with a single dose or orally with the high dose of bacterin. Both of these groups had 100% survival. These results show that a low dose of *Y. ruckeri* bacterin induces full protection when the bacterin is administered anally. Oral vaccination also induces full protection, however, at a dose 50 times higher than if the fish were to be vaccinated anally. This indicates that much of the orally fed antigen is digested in the stomach before it reaches the second segment of the intestine where it can be taken up as immunogenic antigens and presented to lymphocytes.

## Introduction

*Yersinia ruckeri* serotype O1 biotype 1(BT1) causing enteric redmouth disease (ERM) in rainbow trout was initially reported from Hagerman Valley in the US in the 1950's [1]–[3]. Since then, it has been reported from trout producing fish farms around the world [4]. The mortality in ERM infected rainbow trout farms can reach up to 70% in a stock. In order to prevent such devastating outbreaks with ERM, appropriate vaccination and good husbandry is essential [5]–[7]. More recently a *Y. ruckeri* O1 BT2 has been isolated from naïve, as well as ERM vaccinated rainbow trout in several parts of the world [8]–[12].

Bacterial pathogens adhere to and penetrate through mucosal surfaces [13] and one route of entry for *Y. ruckeri* in rainbow trout is known to be the gut mucosa [14]. In rainbow trout, subcutaneous hemorrhages in the mouth and throat are strongly indicative of the disease, hence the term enteric redmouth disease. In infected fish suffering from bacterial hemorrhagic septicemia *Y. ruckeri* can be isolated from almost all organs. The gross pathology of the inflamed lower intestine is one of the most significant clinical diagnostic sign of ERM [15]. The mucosa becomes edematous and necrotic and the lumen is filled with yellow pus containing *Y. ruckeri* and epithelial cells [16]. Chronically infected carriers spread *Y. ruckeri* from the intestine with the feces to the water and thus infect other fish [17]. A model mimicking a natural infection in rainbow trout is available, which makes rainbow trout and *Y. ruckeri* a good host-pathogen model to study the effect of oral vaccination in fish [18].

Successful oral vaccination of rainbow trout against fish pathogenic bacteria has been known for more than 70 years [19]. The first described effective ERM vaccine was an oral vaccine containing a phenol-killed *Y. ruckeri* O1 bacterin [20]. Later it was shown that injection of the bacterin offered better and longer lasting immunity against ERM compared to oral administration [21]. *Y. ruckeri* bacterins can also be used as an immersion vaccine [22]. Immersion is the preferred ERM vaccination method today, because large numbers of small fish can be vaccinated quickly and cheaply and obtains some protection [23]–[25]. The use of *Y. ruckeri* bacterin as an immersion vaccine has brought down the number of ERM outbreaks and losses from the disease. Importantly, it has also increased the growth of vaccinated fish and resulted in diminished use of antibiotics to treat ERM infections [26]. Recently, it was demonstrated that immersion ERM vaccinated rainbow trout develop *Y. ruckeri* specific IgM antibodies in the serum and that these antibodies are protective against the disease [27].

The efficacy of oral fish vaccines have been debated since they were invented. It seems that the effects depend on the gastric transit, the pathogen, as well as the infection model when tested experimentally [28], [29]. Recently, the AquaVac ERM Oral vet. booster vaccine against ERM was tested in an experiment, using a bath infection with *Y. ruckeri* O1 BT 1. Both bath vaccinated and the group that also received an oral booster vaccination showed full protection. Hence, no conclusions regarding the effect of the oral booster vaccination could be drawn [18]. The objective of the present study was to investigate whether oral or anal vaccination can protect rainbow trout against ERM when the vaccines are used for primary and booster vaccination, as well as to understand how these vaccines induce immunity. AquaVac ERM Oral vet. was used for both primary oral and booster vaccination in the present experiment. Furthermore, an experimental *Y. ruckeri* O1 BT1 bacterin with well documented effect as bath vaccine [18], was administered orally in two concentrations, as well as anally in order to avoid gastric degradation. The levels of *Y. ruckeri* specific antibodies in the plasma were detected by enzyme-linked immunosorbent assay (ELISA) in order to clarify the protective immunity, if any. The host-pathogen interaction in the intestine of the different experimental groups was visualized by immunohistochemistry during the infection, suggesting different immune responses and levels of protection depending of the immunization route.

## Materials and Methods

### Ethics Statement

The study was licensed by the National Animal Experimentation Board (license nr. 2012/561-147) according to the EU Directive EU 86/609. The rainbow trout were treated in accordance with the Animal Experimentation Act of Denmark, which is in accordance with the Council of Europe Convention ETS 123.

### Hatching and rearing of pathogen-free rainbow trout

Three hundred rainbow trout were hatched and reared in 500 L fiberglass tanks under pathogen-free conditions (AquaBaltic, Denmark). The pathogen-free status was obtained by hatching certified and disinfected (ActoMar K30) trout eggs in the indoor, recirculated, pathogen-free hatchery. The fish were transported to the experimental facility at the University of Copenhagen at an average body weight of 13.5 g. The pathogen-free status of the fish was confirmed by sampling of some individuals, which were euthanized and analyzed by standard bacteriological methods upon arrival. Further, they were all tested sero-negative for specific antibodies against *Y. ruckeri* O1 BT 1. At the University of Copenhagen the trout were kept in eight 100 L aquaria with continuous aeration and internal bio-filters (1200 L/h. Eheim, Germany). The average water temperature was 15°C, and half of the water was changed every other day. The photoperiod was maintained at a 12 h light and 12 h dark cycle. The fish were hand-fed a commercial trout feed (BioMar, Denmark) 1% relative to the average biomass.

### Commercial oral ERM vaccine

The commercial oral vaccine applied in the present experiment was AquaVac ERM Oral vet (MERCK Animal Health) containing 5×10^8^ CFU/ml formalin inactivated *Y. ruckeri* (Hagerman strain, serotype O1, BT1) [30].

### Production of the experimental oral and anal vaccine

The experimental oral and anal vaccine consisted of a bacterin of formalin-inactivated *Y. ruckeri* in different doses (see table 1).The commercial and experimental oral ERM vaccine were given in the same dose, in order to compare the protective effect of the two vaccines. The *Y. ruckeri* O1 BT1 (strain 392) [8] bacterin used for the experimental oral and anal vaccine was produced as described earlier by Raida et al. 2011 [31]. Briefly, the *Y. ruckeri* bacteria were grown in Luria Bertani broth (LB) (Oxoid LP0042) at 20°C for 36 h and quantified as colony forming units (CFU) by the plate spread method on blood agar plates (State serum Institute, Denmark). The bacteria culture was inactivated by adding 1% formalin (v/v) to the culture followed by a two hour incubation on a plate shaker. The inactivated bacteria were washed 3 times in PBS to remove the formalin. It was confirmed that the bacterin was completely inactivated by plate spreading onto blood agar plates before use. The concentration of the washed bacterin in PBS was adjusted to the approximate concentration given in table 1.

**Table 1:** Vaccination doses used in the experiment.

Group	Primary vaccine dose	Booster vaccine dose
Control	0	0
Control	0	0
AquaVac	1×10^8^ CFU/fish	0
AquaVac w. Boost	1×10^8^ CFU/fish	5×10^7^ CFU/fish
Exp. Oral	1×10^8^ CFU/fish	0
Exp. Oral w. Boost	1×10^8^ CFU/fish	5×10^7^ CFU/fish
Exp. Oral x 50 w. boost	5×10^9^ CFU/fish	5×10^9^ CFU/fish
Exp. Anal	1×10^8^ CFU/fish	5×10^7^ CFU/fish

The table shows the average dose per fish (CFU/ml) of *Y. ruckeri* bacterin applied for vaccination. Some groups were booster vaccinated four months post primary vaccination. Data regarding CFU/ml in AquaVac were supplied by the vaccine manufacturer [30].

### Oral vaccination

Upon arrival, the fish were split into eight groups with 35 fish in each. The groups received either no vaccine (control groups), the commercial or experimental vaccine with or without booster vaccination (see table 1). The oral vaccines were all coated onto 1 mm trout feed pellets (BioMar), which were not top-coated with oil in advance, as usual. This special feed was used to secure that the bacterin vaccine was properly absorbed into the feed, as well as to avoid leakage to the water during feeding. The feed pellets were thoroughly mixed with the bacterin suspended in PBS. After the bacterin was absorbed into the feed pellets, the pellets were top-coated with high quality fish oil to encapsulate the vaccine (BioMar). Feed pellets absorbing the commercial vaccine were not top-coated with oil since the purchased vaccine was mixed with fish oil in advance. This vaccine is intended as a booster vaccine only and administered in a dose of 0.01 ml/fish/day [30]. Therefore, the amount administered to the fish was doubled to 0.02 ml/fish/day when the vaccine was used for primary vaccination. For booster vaccination with AquaVac, the feed pellets were coated with vaccine according to the manufacturer's recommendations. The 35 fish in each tank were fed 1% (w/w) of average bodyweight throughout the experiment. On average each fish received 0.02 ml oral vaccine in feed pr. day from day 1–5, then normal feed without vaccine from day 6–10, and finally vaccine coated feed from day 11–15 (double dose relative to boosting dose). The experimental oral vaccine (Exp. Oral) was given in the same dose as AquaVac (containing 5×10^8^ CFU/ml formalin-inactivated *Y. ruckeri*). One group (Exp. Oral ×50) received a 50 times higher CFU concentration of *Y. ruckeri* bacterin in order to investigate the effect of a high dose on the protection against ERM (Table 1).

The control groups were fed oil top-coated feed pellets like the experimental groups, but without any vaccine added to it (sham-vaccinated).

The booster vaccine was administered in the same manner as the primary vaccine, but each fish received 0.01 ml oral vaccine in feed pr. day, i.e. five days vaccine feed, five days normal feed, followed by another five days with vaccine feed.

### Anal vaccination

In a pilot experiment using euthanized 15 g rainbow trout, 100 μl of Alcian blue was injected anally using a catheter (BusterCat Catheter, sterile, 1.0×130 mm, Cat. N: 273451). This experiment showed that 100 μl was too large a volume, running the risk of the bacterin leaking back out. Consequently, only 50 μl of 2×10^9^ CFU/ml was used for vaccination, so that the administered dose was 1×10^8^ CFU/fish, the same dose as the experimental oral group received during the total primary vaccination period. In order to avoid daily repeated anal re-vaccination that would be stressful to the fish, the vaccine dose was given in just one shot. The anally intubated group was boosted with a single shot of 50 μl vaccine containing 1×10^7^ CFU/fish four month post primary vaccination (Table 1).

**Figure 1:**
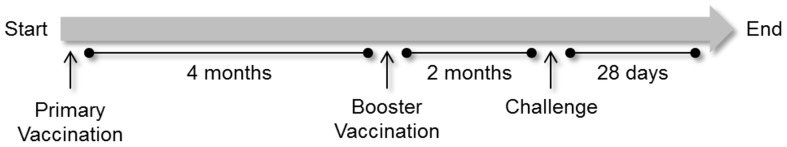
Flow chart showing the experimental setup of vaccination and re-vaccination. All vaccinated groups received a primary vaccination at the start of the experiment. The booster vaccinated groups received a re-vaccination four month post the primary vaccination. All groups were bath challenged with *Y. ruckeri* O1 BT1 six months post primary vaccination and the mortality in the groups were monitored during four weeks.

See figure 1 for an overview of the experimental setup.

### Effect of *in vivo* passage in rainbow trout on the virulence of *Y. ruckeri*

Prior to the final challenge experiment it was attempted to increase the virulence of the strain of *Y. ruckeri* O1 BT 1, by *in vivo* passage of the bacteria in rainbow trout. Shortly, 10 naïve rainbow trout fry were bath infected for one hour, and the bacteria were re-isolated from the head kidney of the first moribund fish. It was confirmed that the bacteria were *Y. ruckeri* O1 BT 1, and the re-isolated *Y. ruckeri* were used to inoculate a new LB broth. Ten naïve fry were bath infected for one hour with the re-isolated *Y. ruckeri*.

### Bath challenge with *Y. ruckeri*

One of the control groups was sham infected in clean water. The other control group as well as the six vaccinated groups of rainbow trout were bath challenged in seven separate 20 L aquaria for seven hours in 5 L water (15°C). The challenge aquaria were continuously aerated and contained 6.33×10^8^ CFU/ml *Y. ruckeri* O1 BT1 (strain 392)[8]. After bath challenge each group was moved back to their respective 100 L aquaria containing clean water. During the 28 days of challenge the tanks were monitored three times daily as a minimum in order to record and humanely euthanize moribund fish. As a chosen humane endpoint, fish were considered moribund when they met one or more of the following clinical criteria: abnormal swimming patterns, loss of equilibrium, isolated behavior combined with a lack of response to feeding. All moribund fish, as well as fish that survived the full length of the challenge experiment, were euthanized by an overdose of MS222 (200 mg/L)(Sigma-Aldrich, Denmark).

### Re-isolation of *Y. ruckeri* from head kidney of moribund rainbow trout

**Figure 2:**
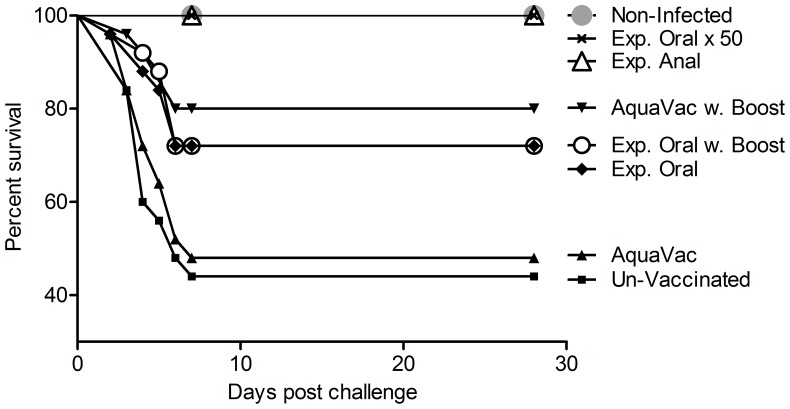
Protective effect of oral and anal vaccination following bath challenge with *Yersinia ruckeri* O1. The figure shows percentage survival of *Y. ruckeri* bath infected rainbow trout six month post primary vaccination. The fish were bath infected in 6.33×10^8^ CFU/ml *Y. ruckeri* O1 BT 1 for seven hours. The highest mortality was seen in un-vaccinated control group (56%) which were significantly higher than the non-infected control group (0%, P<0.0001). The groups that received the high dose of *Y. ruckeri* bacterin orally or the single dose via anal intubation had significantly higher survival than the un-vaccinated control group (P<0.0001 and P<0.0005, respectively). 0% mortality was registered in these groups. Interestingly, there was significantly higher survival in the anal vaccinated group compared to the oral vaccinated group which got the same dose of the *Y. ruckeri* bacterin, but administrated by two different routes (P = 0.028).

Swabs were taken from the head kidney of moribund fish during the challenge experiment (Fig. 2). These swabs were plated onto blood agar plates and incubated for 48 hours. Mortalities were only considered to be caused by *Y. ruckeri* O1, if the bacteria were recovered on the agar plates and showed positive agglutination with specific antibodies (Mono Yr 50 test, Bionor lab, Norway).

### Detection of *Y. ruckeri*-specific IgM in blood plasma with ELISA

Blood samples for measuring the amount of specific antibodies against *Y. ruckeri* by ELISA were taken at three different stages in the experiment: prior to feeding the booster vaccine, pre-challenge, and seven days post bath challenge. Five fish from each group were sampled at each time point and euthanized using an overdose of MS222 (200 mg/L). The weight was recorded for each individual fish and a blood sample was collected from *vena caudalis* using heparinized syringes. Blood samples were immediately stored on ice. The plasma fraction was isolated by centrifugation (10 min, 4000×*g*, 4°C). The obtained plasma samples were stored at −20°C until further analyzed by ELISA.

### ELISA

The ELISA protocol for detecting *Y. ruckeri* specific antibodies in rainbow trout plasma has been described previously by Raida *et al*. [18] and was used with minor modifications. Briefly, 96-well ELISA-plates (NUNC MaxiSorp) were coated with sonicated *Y. ruckeri* O1 BT1 (strain 392) in an alternating pattern, coating odd columns with antigen, but keeping even columns free of antigen. Subsequently, all wells were blocked using a carbonate-bicarbonate blocking buffer (Sigma-Aldrich, Denmark). This coating pattern was used to enable correction for sample-specific differences in plastic binding tendencies. The plates were then sealed and kept at -20°C until needed. Based on optimizing pilot work, a fixed dilution of 1∶25 was chosen for the ELISA setup. All dilutions were made in an assay diluent as described in Raida *et al*. [18]. All the plasma samples were tested in triplicate pairs of antigen coated and -uncoated wells. A positive plasma sample with a high titer of anti-*Y. ruckeri* antibody levels was included on every plate for interplate calibration. As an additional control measure, wells containing only the assay diluent were included on each plate, to allow for plate-specific correction for background. All samples were incubated on the plate overnight at 4°C. Unless otherwise stated, all subsequent incubations were at room temperature and all washing steps were performed as three washes with washing buffer. After sample incubation, plates were washed and mouse-anti-salmonid immunoglobulin (Ig) (AbD Serotec, 1∶500 dilution in assay buffer) was added to each well to incubate for 1 h. After a subsequent wash, an HRP-conjugated rabbit-anti-mouse Ig (AbD Serotec, 1∶500 dilution in assay buffer) was added and left to incubate for 1 h. The plates were then washed, and 100 μl tetramethylbenzidine substrate (Sigma-Aldrich; Denmark) was added to each well, after which they were placed on plate shaker, and the reaction was observed for 5-10 minutes. The reaction was stopped by the addition of HCl to a final concentration of 0.5 M, and the plates were analyzed immediately using a plate-reader (Epoch, BioTek Instruments, Inc.)

**Figure 3:**
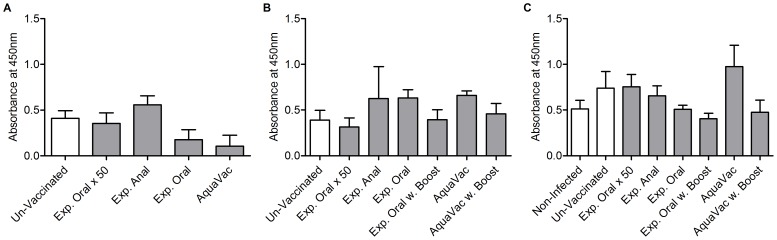
Detection of *Y. ruckeri* specific antibodies in plasma. Plasma samples were collected A) Pre-booster vaccination B) Pre-challenge infection and C) seven days post infection. No statistical significances in the antibody levels were found among all groups at any of the sampling time points.

Since the samples were analyzed at a relatively low dilution, the final analysis were performed twice, using either the sample-specific background obtained from uncoated wells, or the plate-specific background measurements obtained from the antigen coated wells incubated with assay diluent instead of sample. All data shown and analyzed in figure 3 are based on the former method (individual, sample-specific background correction).

### Sampling of intestine and paraffin imbedding for histology

Samples were taken from the second segment of the mid-intestine of the hindgut which contains the M-like cells in rainbow trout as described by Fuglem *et al* [32]. Tissue was collected from three rainbow trout per group, seven days post infection. The samples were preserved separately in 4% buffered formalin for 24 hours and then transferred to 70% ethanol for storage until further processing for paraffin embedding. The tissue was paraffin embedded as described by Chettri *et al*. [33] with minor modifications. Briefly, the samples were dehydrated in increasing concentrations of ethanol (70%, 96% and 99%), then cleared in xylene and finally embedded in liquid paraffin. The paraffin blocks were sectioned in 4 μm thick sections on a microtome (Leica Microsystems). Paraffin sections were transferred to a water bath (40°C), mounted on glass slides (SuperFrost Ultra Plus, Menzel-Glaser) and dried overnight at 40°C. The slides were then kept at 4°C until further processing.

### Immunohistochemistry with polyclonal anti-*Y. ruckeri* antibodies

*Y. ruckeri* bacteria in the intestine sections were stained with a rabbit-anti-*Y. ruckeri* O1 BT 1 polyclonal antiserum, recently characterized and described by Chettri et al. [33]. Pre immune serum from the same rabbit was used as negative control to validate the specificity of the antiserum.

The sections were deparaffinated with xylene and rehydrated to water through series of baths with decreasing ethanol concentration (99%, 96% and 70%) and then incubated with 1.5% H_2_O_2_ in Tris-buffered saline (TBS) (Dako, Denmark) for 10 minuttes to quench endogenous peroxidase activity. Contrary to the work performed by Chettri *et al*., no heat-induced epitope retrieval was performed. Before the addition of antiserum, each slide was covered in 2% bovine serum albumin (BSA) for 10 minutes to prevent non-specific antibody interaction. The slides were then incubated overnight at 4°C in a 1∶10.000 dilution (in TBS +1% BSA) of anti-*Y. ruckeri* antiserum. Subsequently, the slides were gently washed in TBS, covered in an HRP-conjugated goat-anti-rabbit antibody polymer construct (Dako EnVision+) and incubated for 30 minutes at room temperature. After incubation, the sections were washed and processed in an aminoethylcarbazole solution. After a subsequent wash, slides were counterstained in Mayer's haematoxylin, and mounted with coverslips.

### Statistical analysis

All statistical tests were carried out using GraphPad Prism 5 (GraphPad Software, Inc. San Diego, USA). A significance level of 5% was applied in all tests. Mortality data obtained during the challenge experiments were analyzed using the Kaplan-Meier test, to test for differences in mortality between the groups. One-way analysis of variance and Tukey's Multiple Comparison Test as post test was used to test for potential difference in average weight between the groups before challenge. The results from the ELISA analysis failed the Kolmogorov-Smirnov test for normality. Hence, they were analyzed by the Kruskal-Wallis method, using Dunn's multiple comparison test in order to identify possible differences in the antibody levels between groups.

## Results

### Fish

The rainbow trout increased in average weight from 13.5 g to 35.6 g during the experimental period. There were no significant differences in weight between the eight groups at the time of challenge (p = 0.16). Due to a drop in air pressure in one aquarium, 10 fish in the Exp. anally vaccinated group were lost prior to challenge. No mortality was observed in any of the other vaccinated groups during the six months, between the primary vaccination and the challenge experiment.

### Effect of *in vivo* passage on the virulence of *Y. ruckeri*

No increase in the mortality was detected after *in vivo* passage of *Y. ruckeri* serotype O1 BT1 in rainbow trout (data not shown). Since there was no increase in virulence after passing the bacteria through fish, the original isolate kept in glycerol at minus 80°C was used to inoculate the LB broth for the final challenge experiment.

### Challenge results

The bath challenge induced significant mortality in the un-vaccinated control group (56% mortality) compared to the non-infected control group (P<0.0001, Fig. 2). The onset of mortality started two days post infection and peaked on day four. The mortality ceased at day eight post bath infection.

There was no significant increase in survival in the group vaccinated with the primary AquaVac compared to the group of un-vaccinated infected control fish (P = 0.75). AquaVac vaccinated fish, which also received an oral booster vaccination, showed significantly increased survival relative to control (P = 0.006). There was a significant increase in survival rate due to the booster vaccination with AquaVac (P = 0.015).

Both experimental vaccines, given in the same dose as the AquaVac vaccine, induced significant protection compared to the un-vaccinated control group (Exp. Oral P = 0.036 and Exp. Oral w. booster P = 0.024). Booster vaccination with the exp. oral vaccine did not improve the survival rate (P = 0.94).

There was no significant difference in survival rates between the groups that had only received a primary oral vaccination AquaVac (52% mortality) and Exp. Oral vaccine (28% mortality) (P = 0.075). Further, no significant difference in the protective effect was observed among the AquaVac and Exp. Oral vaccinated group, when they had been booster vaccinated four months post the primary oral vaccination (P = 0.53).

Both the Exp. Oral ×50 and the Exp. Anal vaccinated group showed full protection, which is highly significant relative to the un-vaccinated control group (P<0.0001 and P = 0.0005, respectively).

The Exp. Oral ×50 vaccinated group was significantly better protected against ERM compared to all other oral vaccinated groups (AquaVac P<0.0001, AquaVac w. booster P = 0.02, Exp. Oral P = 0.006 and Exp. Oral w. booster P = 0.006).

Since both the Exp. Oral ×50 vaccinated group and the Exp. Anal vaccinated group showed full protection, there was no difference in survival between those groups (P = 1).

Interestingly, there was a significantly increased survival in the Exp. Anal vaccinated group compared to the Exp. Oral (w. booster) vaccinated group both having received the same dose of experimental bacterin, however administrated by two different routes (P = 0.027).

### Re-isolation of *Y. ruckeri* from moribund fish

In order to determine the specific cause of death, samples collected from the head kidney of moribund fish were examined for *Y. ruckeri* after 48 h incubation. Bacteria were recovered from fish in all groups that showed mortality. The bacteria formed white non-hemolytic colonies on blood agar. They were diagnosed as *Y. ruckeri* by use of a specific agglutination kit containing specific polyclonal anti *Y. ruckeri* antibodies. The isolated *Y. ruckeri* were further tested positive for hydrolysis of Tween 80, as well as motility under a microscope, identifying the strain as biotype 1. The moribund fish showed classic signs of ERM infection including subcutaneous hemorrhages in the mouth, bilateral exophthalmos, petechial hemorrhages in the internal organs, sideline, eyes, around the dorsal fins and the anus. Furthermore, moribund fish were anorexic, turned dark and showed isolated behavior, often found swimming near the surface.

### Detection of *Y. ruckeri*-specific IgM in plasma

Plasma samples collected at various stages during the experiment (pre booster vaccination, pre challenge and seven days post challenge) were analyzed for specific anti-*Y. ruckeri* antibodies. Figure 3 A and B show the ELISA results from samples collected prior to the infection, and C shows the results obtained seven days post infection, where mortality had been high within the groups that were not protected. Although antibody levels increased slightly in some groups during the infection, no significant differences were detected between any of the groups at each time point.

The initial optimization of the ELISA setup favored the use of a relatively low degree of dilution of each sample (25 times). Since this means that plasma proteins will potentially be present in concentrations that could affect the outcome of the ELISA, the obtained data were analyzed using both a sample-specific and a plate-specific background measurement. However, it was found that the general patterns seen in the results were very similar, and that there were no differences in the results of the statistical analysis. The sample-specific background was chosen for data analysis (Fig. 3).

### Immunohistochemistry for detection of *Y. ruckeri* in the intestine

**Figure 4:**
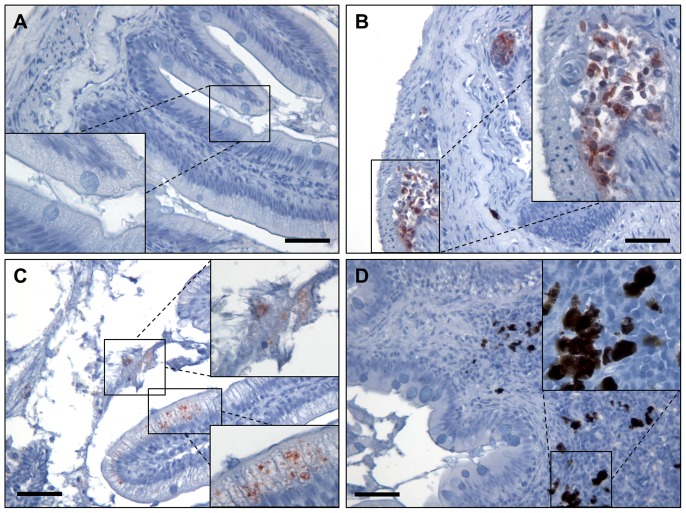
Immunohistochemical detection of *Y. ruckeri* O1 in the second segment of the mid-intestine. *Y. ruckeri* bacteria in the intestine were stained specific with polyclonal rabbit-anti *Y. ruckeri* antibodies. Samples for immunohistochemistry were collected seven days post infection, where the mortality rate is different between the groups due to dissimilar levels of immunity. (A) No *Y. ruckeri* were present in the un-infected control group. (B) In the infected un-vaccinated group *Y. ruckeri* were found in large amount in the blood vessels in the sub-mucosa. Large amount of *Y. ruckeri* was also seen inside monocytes. (C) In the well protected orally vaccinated group (50 x dose) *Y. ruckeri* were found in the lumen of the intestine, as well as in intestinal epithelial cells. However, no bacteria were observed in the sub-mucosa, indicating some level of protective immunity. (D) In the full protected anally vaccinated group only few *Y. ruckeri* bacteria could be detected, indicating a high level of protective immunity and clearance of the infection. Bar = 50 μm. 200× magnification.

No *Y. ruckeri* O1 BT1 bacteria were found in the intestine of non-infected control fish (Figure 4 A) and no pathological changes were observed in these fish. In the sections from un-vaccinated *Y. ruckeri* infected fish high numbers of *Y. ruckeri* present mainly in the blood vessels in the intestinal tissue, showing systemic infection. Monocytes containing *Y. ruckeri* was observed in the blood vessels (Figure 4 B). It is not known whether *Y. ruckeri* has infected the trout through the intestine or another route.

In the Exp. Oral ×50 vaccinated group (0% mortality during challenge) *Y. ruckeri* was found to be present in the epithelial cells, as well as in mucus in the lumen. However, the bacteria were not found deeper in the tissue, or in the blood vessels. Compared to the infected control group it seems the bacteria cells are contained within the epithelial cells, probably due a protective immune mechanism.

In the intestine sections of fish from the Exp. Anal vaccinated group only very few *Y. ruckeri* bacteria were found. Interestingly, many melanomacrophages were found in the sub-mucosa in these fish. In addition to this, aggregates of leucocytes containing lymphocytes were found in the intestinal tissue. Likely, these immune cells have eradicated the *Y. ruckeri* bacteria at an early time point post infection and thereby prevented spreading of the bacteria from the lumen of the intestine to the blood vessels (Figure 4 D).

## Discussion

The gastrointestinal tract is the prime site for absorption of nutrients and fluids, but also serves as a barrier between the host and the external environment where both commensal and pathogenic microorganisms are present. When entero-invasive bacterial pathogens trespass the intestinal epithelium causing rupture and inflammation, the innate and adaptive immune system are both activated [13]. In accordance to this, *Y. ruckeri* infection has been shown to trigger both innate and adaptive immune responses in intestinal epithelial cells in rainbow trout [34].

Vaccination is a keystone in prophylactic strategies preventing outbreaks of fish pathogenic bacterial diseases in aquaculture. The first commercial fish vaccine consisted of a bacterin of *Y. ruckeri* serotype O1 BT 1. This vaccine has been very successful and has now been used for more than 35 years. Vast experience has been gained regarding the applications of the vaccine that can be utilized through several mucosal immunization routes such as bath, oral and anal application, all resulting in significantly increased survival compared to un-vaccinated control groups in challenge experiments [21], [35], [36].

The potential of oral vaccination of fish has been met with mixed opinions [28], [37], [38]. This is due to the fact that different vaccines have provided variable durations and levels of protection against different pathogens under different circumstances. One difficulty with oral vaccination is to determine exactly how much of the vaccine is taken up by each fish. Therefore, different levels of protection within the same group of fish are a possibility. In addition, antigens must travel to the second segment of the hindgut in order to be taken up, which has previously been proposed to be the primary hindrance in maximizing oral vaccination efficacy [32], [39]. One could speculate that in oral vaccines with a low antigen concentration the majority of these antigens are broken down in the fish stomach, whereby their delivery to the M-cell-like cells in the distal part of the intestine is prevented [32].

The high concentration of killed *Y. ruckeri* applied in the Exp. Oral (50x dose) vaccinated group in the present study would not be financially feasible for mass production of oral vaccines because of the vast amounts of broth required for bacteria growth. However, it demonstrates that oral vaccination of rainbow trout is possible, which brings new optimism into the field with the possibility of minimizing losses due to *Y. ruckeri* infections, while keeping labor costs and efforts to a minimum. Nevertheless, other reasons for inefficient delivery of oral bacterin could exist such as the possibility of breakdown of the bacterin in the feed, or loss of the protective coating, once the feed comes into contact with water.

Thus, future perspectives of oral vaccination against *Y. ruckeri* will rely heavily on research focusing on delivery methods of the inactivated bacteria to the hindgut of the fish, containing the mammalian M-like cells [32]. It is suggested that protection of the antigens during gastric passage might be achievable by microencapsulation of the antigens.

The benefits of effective oral vaccines for fish are obvious: it will be easy to vaccinate large numbers of fish in a short time, and the fish become less stressed compared to handling for vaccine injection. Furthermore, the oral vaccine can be used for all sizes of fish, it is inexpensive and booster vaccination can be conducted as often as needed without causing extra effort. Regarding the effect of oral ERM vaccines, not much is known. Recently, it has been demonstrated that ERM bath vaccination increases the *Y. ruckeri* specific antibody level in the blood of rainbow trout [31], and another study has shown that transfer of even low amount of *Y. ruckeri* specific IgM to naïve trout passes the immunity [27]. The anal vaccination route was attempted because of the recent discovery of M-like cells in the second segment of the mid intestine, as well as to bypass the stomach, hypothetically the cause for bacterin breakdown resulting in unreliable development of immunity against infections. In the present study, the anal vaccinated group received the same amount of bacterin as the experimental oral vaccination groups, however in a single dose since vaccinating the fish anally at a daily basis with a lower dose would have caused unnecessary stress to the fish. In terms of protection, anal intubation of the bacterin resulted in a higher level compared to oral delivery of the same dose of bacterin. This suggests that the immunizing antigens are destroyed in the stomach [40] and further indicates that the distal intestine may play a central role in antigen uptake. It is thus likely that immunogenic antigens are taken up from the lumen of the gut in rainbow trout and presented to lymphocytes.

The challenge infection showed to be successful and resulted in 56% mortality in the infected un-vaccinated control group, closely followed by the AquaVac group (52% mortality). In addition there were no differences in survival rates between the boosted and non-boosted groups (28% mortality in both groups) which received the experimental vaccine (same dose as the commercial vaccine). There was a significant effect of boosting with the commercial AquaVac vaccine, but that was mainly due to a very low survival rate in the group that had only received the primary oral vaccination.

Interestingly, there were no significant differences in the protective effect between the Exp. Oral vaccine and AquaVac Oral vaccine, with or without booster vaccination. This finding is interesting because the commercial vaccine contains a proprietary antigen protection vehicle, designed to protect the antigens from being degraded when passing through the stomach.

The experimental vaccine confers significantly higher protection when it is applied anally compared to orally in same concentrations. It is suggested that this change is due to a breakdown of some of the antigens in the stomach of the orally vaccinated rainbow trout. This reduced effect of the orally applied experimental vaccine could be defeated by increasing the dose of antigens 50 times, which further indicates that the amount of antigen that reaches the distal intestine is crucial for the development of protection against ERM. The positive effect of oral ERM vaccination has also been documented in another study, where an intraperitoneal challenge model was applied [41]. In the present study it was demonstrated that the experimental oral vaccine induces protective immunity but that the dose has to be higher than recommended for the commercial oral ERM vaccine. These results correlate with the results obtained by Gravningen et al. who found a correlation between increasing doses of *Y. ruckeri* O1 bacterin in the feed and the protective effect against ERM in rainbow trout [41]. Injection of pathogenic bacteria is an often used infection model applied for evaluation of the effect of fish vaccines. The ability to document the exact amount of bacteria administered to each fish makes it appealing in the effort to eliminate potentially variable parameters [42]. However, this method unfortunately also bypasses the external mucus layers of the fish and thereby the mucosal immune defense mechanism, found in skin, gills, as well as intestine. Recent research has shown that the protective effect of the ERM immersion vaccine was depending on the infection model applied for evaluation [43]. It is therefore important that waterborne infection models are applied in order to evaluate the protective effect of mucosal vaccines such as oral, anal and immersion vaccines. In the present work we have successfully used a bath infection model which mimics a natural *Y. ruckeri* infection and obtained a mortality of more than 50% in the un-vaccinated control group.

It has previous been shown by Johnson and Amend that anal vaccination against ERM in trout confers better protection than oral or immersion vaccination, based on the same *Y. ruckeri* bacterin [35]. Further, they found that there was no difference in the protection between ERM immersion and oral vaccination [35]. Several studies where bacterins were administered by immersion have shown that the bacterin is taken up in the intestine. A significant amount of the bacterin is detected in the intestine of the fish after immersion vaccination, and the bacterin persists in the gut [44]–[46]. Antigens from orally vaccinated Atlantic salmon was found to be present in the head kidney up to six weeks post vaccination and in the spleen up to 16 weeks post vaccination clearly demonstrating a systemic uptake [47].

The significantly reduced mortality in the group that received an oral booster with the AquaVac vaccine in comparison to the respective non-boosted AquaVac group shows the protective benefit of oral booster vaccination four months post-primary vaccination. It is well known that booster vaccination enhances the scale and specificity of the immune response. When the antigen is presented to previously primed leucocytes, they are stimulated to proliferate. Again, it should be stated that the AquaVac ERM oral vaccine is only recommended as a booster vaccine by the manufacturer [30], applied to the fish four to six months post immersion vaccination.

The full protection against ERM gained in the present study with the anally vaccinated group is in agreement with the better protection seen in other salmonids against ERM and vibriosis after anal intubation in comparison to oral and bath vaccination [35]. In the present study the anally vaccinated group showed 100% survival relative to 44% survival in the un-vaccinated group. Full protection was obtained in both orally and anally vaccinated rainbow trout, but no increases in *Y. ruckeri* specific antibodies were detected in the plasma samples collected from these groups, compared to un-vaccinated control groups. Surprisingly, the level of *Y. ruckeri* specific antibodies was unaffected by oral and anal vaccination, when it has been shown that rainbow trout bath vaccinated with the same experimental vaccine showed low but significantly increased levels of anti *Y. ruckeri* IgM [18]. The present finding indicates that the protective immunity induced by the vaccine is different and depend on the mucosal route of immunization. The results from the present study agree well with those of [40] who found that rainbow trout orally vaccinated with *Y. ruckeri* bacterin were protected against ERM but did not develop agglutinating antibodies in plasma against *Y. ruckeri* [40]. Oral vaccination of Atlantic salmon against *Piscirickettsia salmonis* has been shown to be very effective. The effect is due to both a systemic and a local intestinal specific antibody response which protects the vaccinated fish [39]. The water temperature is important for development of antibodies post vaccination [43] and in the present study the fish were kept at an average water temperature of 15°C, which is near the optimal thriving temperature for rainbow trout [48].

The protective mechanism behind the immunity induced by oral and anal immunization is at present unknown, and the vaccine is likely taken up by the M-like cells in the lower intestine. It is therefore suggested that oral and anal vaccination induce a local immunity in the intestine due to activation of gut-associated lymphoid tissue (GALT) associated with the gastrointestinal tract in vaccinated trout. Antigen sampling cells in connection with lymphocytes has previously been described in the intestine of salmonids and is believed to be an equivalent to or an early evolutionary precursor of GALT in fish [32]. The rainbow trout intestine is known to take up *Y. ruckeri* O1 bacterin, but the uptake mechanism is unknown [49]. The external mucosal layer of fish is the first physical barrier that comes into contact with the surrounding water containing various pathogenic microorganisms. This layer protects the fish against invasion of bacteria and is constantly sloughed off, which prevents bacteria from attaching and thereby invading the underlying epithelium [50]. The mucous layer plays a crucial role in disease resistance which can be enhanced by vaccination [51]. Likewise, the intestine is protected by a mucus layer, which has been shown to contain antibodies [50]. Oral vaccination of rainbow trout with bacterin induced significant amounts of antibody-secreting B-lymphocytes in the intestinal epithelium eight weeks post vaccination, demonstrating an active role of the intestine in antibody secretion in rainbow trout [51]. These results correlates well with the increased protection seen in both oral and anal vaccinated trout in the current study, and the increased amount of lymphocytes seen in the intestinal epithelial of the anal vaccinated group. This kind of immunity demonstrates that the intestine is able to mount an immune response in a manner similar to that of other immunologically important tissue, i.e. spleen and head kidney [34].

It has been shown, that the mucosal antibody response in anally immunized trout consist of heterogeneous forms of Ig differing from serum Ig [52]. Recently, IgT was discovered in rainbow trout [53]. IgT is predominantly located in the gut mucus, where it binds to pathogens and gut micro flora [54]. Recently, transcription data obtained from the intestine of naïve trout infected with *Y. ruckeri* has shown increased transcription of IgT and IgM, which was correlated with the systemic amount of *Y. ruckeri* in the infected fish [34]. The results from immunohistochemistry on sections of the second segment of the mid-intestine showed major differences in the amount as well as the localization of *Y. ruckeri* within the intestine. The samples were collected seven days post infection, where the mortality was high in the un-protected groups. This is an interesting time point because different infection patterns between protected and un-protected groups are expected due to the difference in vaccine induced immune responses. *Y. ruckeri* bacteria were found extracellularly and inside monocytes in the blood vessels in the intestinal sub mucosa of the un-vaccinated fish (Fig. 4B) indicating that the bacteria are able to transgress the epithelium of the intestine and cause systemic infection. Surprisingly, a high number of *Y. ruckeri* was found in mucosa in the well-protected orally vaccinated trout (high dose of bacterin), but even more interesting, the bacteria were only found in the epithelial cells of the mucosa indicating that some kind of immune response is preventing them from penetrating deeper into the tissue. Further, *Y. ruckeri* were found associated with the mucus in the intestinal lumen. It is speculated that this can be due to the presence of *Y. ruckeri* specific IgT in the mucus layer. The fact that oral vaccination does not prevent *Y. ruckeri* from infecting the epithelial cells but contributes to the clearance of the infection, has previously been described in ERM bath vaccinated trout [18]. Only few *Y. ruckeri* bacteria were found in intestine of anally vaccinated rainbow trout which is likely due to a stronger immune response in this group compared with the other infected groups.

No mortality was registered in the vaccinated and highly protected groups. In future work, the potential involvement of *Y. ruckeri* specific IgT antibodies in the protective immunity will be investigated. Furthermore, future research will be focused on investigation of the minimum dosage of bacterin administered via the feed inducing proper immunity in rainbow trout. It could be that lower bacterin concentrations can also induce sufficient protection if the antigens are coated to avoid digestion, thereby making this a considerably more viable solution, from a financial and manufacturing standpoint. Furthermore, with successful commercialization of oral vaccines, feed manufacturers could produce fish feed already coated with bacterin, thereby making primary and booster vaccination of fish considerably easier and open up for a new niche in fish feed production. The development of an efficient and consistent oral vaccine against ERM would drastically lower the labor demands on fish farms, as well as pave the way for lower antibiotics use and more sustainable aquaculture.
